# Ezetimibe and Cancer: Is There a Connection?

**DOI:** 10.3389/fphar.2022.831657

**Published:** 2022-07-18

**Authors:** Jia Gu, Neng Zhu, Hong-Fang Li, Chan-Juan Zhang, Yong-Zhen Gong, Duan-Fang Liao, Li Qin

**Affiliations:** ^1^ Laboratory of Stem Cell Regulation With Chinese Medicine and its Application, HunanUniversity of Chinese Medicine, Changsha, China; ^2^ Department of Urology, The First Hospital of Hunan University of Chinese Medicine, Changsha, China; ^3^ Institutional Key Laboratory of Vascular Biology and Translational Medicine in Hunan Province, Changsha, China; ^4^ Hunan Province Engineering Research Center of Bioactive Substance Discovery of Traditional Chinese Medicine, Hunan University of Chinese Medicine, Changsha, China

**Keywords:** ezetimibe, NPC1L1, cholesterol, hypercholesteremia, cancer

## Abstract

The high level of serum cholesterol caused by the excessive absorption of cholesterol can lead to hypercholesteremia, thus promoting the occurrence and development of cancer. Ezetimibe is a drug that reduces cholesterol absorption and has been widely used for the treatment of patients with high circulating cholesterol levels for many years. Mechanistically, ezetimibe works by binding to NPC1L1, which is a key mediator of cholesterol absorption. Accumulating data from preclinical models have shown that ezetimibe alone could inhibit the development and progression of cancer through a variety of mechanisms, including anti-angiogenesis, stem cell suppression, anti-inflammation, immune enhancement and anti-proliferation. In the past decade, there has been heated discussion on whether ezetimibe combined with statins will increase the risk of cancer. At present, more and more evidence shows that ezetimibe does not increase the risk of cancers, which supports the role of ezetimibe in anti-cancer. In this review, we discussed the latest progress in the anti-cancer properties of ezetimibe and elucidated its underlying molecular mechanisms. Finally, we highlighted the potential of ezetimibe as a therapeutic agent in future cancer treatment and prevention.

## Introduction

Almost all mammalian cells can synthesize cholesterol. About 50% of total synthesis takes place in the human liver, but most cells cannot completely decompose cholesterol. They can excrete excess cholesterol or store it in lipid droplets as esterified cholesterol esters (Luo et al., 2020). One of the basic excretion pathways of cholesterol is to convert of cholesterol into bile acids through the liver and excrete them to the intestine together with bile, which is a key to maintaining the balance of cholesterol level in the body. Besides *de novo* synthesis and hepatic excretion, intestinal absorption of exogenous cholesterol from the diet plays a vital role in maintaining cholesterol homeostasis (Baigent, 2015). However, the long-term excessive absorption of cholesterol will ultimately lead to many diseases, notably hypercholesteremia (Singh et al., 2013; Aljenedil et al., 2018), which is considered to be a cause of cardiovascular disease (CVD) (Giugliano et al., 2017; Schmidt et al., 2017) and cancer (Nelson et al., 2013; Zhang et al., 2021).

Ezetimibe is an effective cholesterol absorption inhibitor. It can effectively reduce the level of serum cholesterol by blocking the sterol transporter Niemann-Pick C1-Like 1 (NPC1L1). NPC1L1 is a key regulator of cholesterol uptake in intestine cavity through clathrin-mediated endocytosis (Luo et al., 2020). NPC1L1 is expressed exclusively in human liver and small intestine, and is specifically expressed in rodent intestine (Kim et al., 2017). NPC1L1 is mainly localized in the apical membrane of small intestine epithelial cells and hepatic tubular membrane (Jia et al., 2011). NPC1L1 is not only involved in regulating intestinal cholesterol absorption from daily diet, but also modulates hepatobiliary cholesterol excretion by transporting bile cholesterol to hepatocytes (Park, 2013). The cholesterol absorption block caused by NPC1L1 inhibition suppresses the delivery of cholesterol to the liver, leading to the reduction of liver cholesterol storage, promoting liver low-density lipoprotein (LDL) uptake and plasma LDL cholesterol reduction (Preiss et al., 2020). NPC1L1-mediated cholesterol absorption exceeds LDL receptor (LDLR)-mediated hepatic endocytosis, resulting in excessive accumulation of LDL-C, which can be oxidized and transformed into oxidized LDL (ox-LDL). In addition, other cholesterol-lowering drugs, such as statins and proprotein convertase subtilisin/kexin type 9 (PCSK9) inhibitors, are often used. Statins are the earliest used lipid-lowering drugs, which play a role by inhibiting HMG-CoA reductase (HMGCR), the rate limiting enzyme of cholesterol synthesis. PCSK9 inhibitors play a role by binding to LDLR and impairing its function of transporting cholesterol to the liver.

Clinically, ezetimibe has been recommended in the current guidelines and is widely used in the treatment of hypercholesterolemia alone or in combination with statins to prevent adverse cardiovascular disease events and mortality (Ouchi et al., 2019; Mourikis et al., 2020). Epidemiologic studies have shown that the use of statin can reduce the risk of cancer and is conductive to the prognosis of cancer (Walker et al., 2015; Voorneveld et al., 2017). Although no dedicated studies have described the relationship between ezetimibe and the risk of cancer or the prognosis of cancer, it is reported that the high expression of NPC1L1, the target of ezetimibe, is associated with the development and prognosis of colorectal cancer, indicating the potential of ezetimibe in the treatment and prevention of cancer (Kwon et al., 2021). Here, we comprehensively review the existing evidence that supports the role of ezetimibe in cancer chemoprevention and therapy. Next, we will focus on cell-based and animal-based pre-clinical studies to summarize the potential molecular mechanism of the inhibitory effects of ezetimibe in cancer pathogenesis and progression. Moreover, we also discuss whether the controversy over the combination of ezetimibe and statins is related to the increased risk of cancer, and emphasized the potential of ezetimibe as a personalized cancer treatment drug in the future.

## History of Ezetimibe

A series of landmark discoveries have provided people with the latest understanding of ezetimibe and its cholesterol-lowering mechanism. SCH48461 is a potential cholesterol-lowering intestinal absorption substance. Davis group (Van Heek et al., 1997) unexpectedly found that SCH58235, one of the metabolites of SCH48461, displayed a better cholesterol-lowering effect than SCH48461 itself. SCH48461 inhibited cholesterol absorption by 70%, while SCH58235 in bile inhibited cholesterol absorption by more than 95%. Several subsequent studies are also documented (Zaks and Dodds, 1998; Reiss et al., 1999). In 2000, Margaret van Heek and colleagues (van Heek et al., 2000) named SCH58235 “ezetimibe”, and its molecular structure has been well known to researchers. Compared with statins, which mainly inhibit the activity of HMG-CoA reductase, ezetimibe can inhibit cholesterol absorption in the intestine cavity through some other pathway. In 2004, Klett and Patel (Klett et al., 2004) confirmed that ezetimibe specifically binds to NPC1L1 and inhibits its activity.

## Cholesterol-Lowering Mechanism of Ezetimibe

NPC1L1 was first identified because of its high sequence homology with Niemann–Pick type C1 (NPC1) (42% identity and 51% similarity), so it was named NPC1L1. Under normal growth conditions, NPC1L1 mainly exists in the endocytic recycling compartment (ERC). After cholesterol depletion, NPC1L1 rapidly transfers to the plasma membrane (Yu et al., 2006; Ge et al., 2008). Cholesterol supplementation triggers NPC1L1 and cholesterol transport from the plasma membrane to the ERC (Ge et al., 2008). Mechanistically, NPC1L1 interacts with cholesterol on the surface and flotillin at the inner leaflet of the plasma membrane (Ge et al., 2011; Zhang et al., 2011). The combination of Cholesterol and NPC1L1 promotes the formation of specialized membrane microregions rich in cholesterol, flotillins, and gangliosides (Ge et al., 2011; Zhang et al., 2011; Nihei et al., 2018), and leads to the separation of the NPC1L1 C- terminal tail from plasma membrane. Therefore, YVNxxF sequence can be used for NUMB recognition (Li et al., 2014). As a clathrin adaptor protein, NUMB further recruits clathrin and clathrin adaptor AP2 to the invaginated microregions, generates encapsulated vesicles. Then the endocytic vesicles migrate along actin filaments to the ERC (Ge et al., 2008). Bao-Liang Song’s research group recently found that a rare frameshift variant of LIMA1 gene in a Chinese family of Kazakh ethnicity inherited low levels of LDL cholesterol (LDL-C) and reduced cholesterol absorption (Zhang et al., 2018a). LIMA1 connects NPC1L1 to a transportation complex containing myosin Vitamin B (Vb) and facilities cholesterol absorption.

## The Potential of Ezetimibe in Cancer Treatment and Prevention

Over the past decade, many preclinical and clinical studies have reinforced the hypothesis that ezetimibe has the potential to treat and prevent cancer. Interestingly, but unfortunately, the hypothesis that ezetimibe in combination with statins increases cancer risk has aroused great public interest and controversy (Rossebø et al., 2008). Clinical studies show that the cholesterol-lowering treatment by five-year usage of simvastatin does not affect the incidence of cancer and mortality. Therefore, the short-term cancerogenic effects of simvastatin are excluded (Strandberg et al., 2004). So far, in order to understand the effect of simvastatin, a combination of drugs with better cholesterol-lowering effects cannot be ignored. It is reassuring that more and more evidence does not support above hypothesis (Peto et al., 2008; Green et al., 2014). On the contrary, more and more evidence supports that ezetimibe plays an active role in combating cancer. For example, several *in vitro and in vivo* studies (Table 1) show that ezetimibe can inhibit multiple cancers, such as prostate cancer (Solomon et al., 2009), breast cancer (Pelton et al., 2014), pancreatic cancer (Nicolle et al., 2017), urinary bladder cancer (Yang et al., 2021), colorectal cancer (He et al., 2015), hepatocellular carcinoma (Ribas et al., 2021), melanoma (Wang et al., 2022) and renal cell carcinoma (Wang et al., 2022) through various mechanisms, including anti-angiogenesis, apoptosis, anti-proliferation, anti-inflammation, stem cell inhibition, and immune enhancement. Existing evidence shows that ezetimibe should be considered as a safe and effective cholesterol-lowering agent, and may become an academic bomb-shell for cancer treatment and prevention. However, the specific anti-cancer molecular mechanisms of ezetimibe remain to be further clarified and studied.

**TABLE 1:** *In vitro* and *in vivo* evidence of ezetimibe against cancer.

Cancer types	*In Vivo*	*In Vitro*	Mechanisms	Dose and route of administration	References
Prostate cancer	PTEN-null mice	NA	Androgen↓, Ki67↓, TUNEL H score↑	30 mg/kg/day HFHC, p.o	Allott et al., (2018)
	LNCaP cell-derived xenograft mouse models	NA	Ki67↓, TUNEL H score↑, CD31↓, Caveolin-1↓, fibroblast↓, TSP-1↑, SMA↑	30 mg/kg/day HFHC, p.o	Solomon et al., (2009)
	RM1 cell-derived xenograft mouse models, mTORC2−/− mice	NA	Akt↓, mTORC2↓, CPT1A↑, CD8^+^ lymphocyte↑	30 mg/kg/day	Wang et al., (2022)
				alone, p.o	
Urinary bladder cancer	T24 cell-derived xenograft mouse models	NA	Nanog↓, CD44↓, KLF4↓, ALDH1A1↓, ox-LDL induced-CD36/JAK2/STAT3 axis↓	30 mg/kg/day HFHC, p.o	Yang et al., (2021)
Breast cancer	MDA-MB-231 cell-derived xenograft mouse models	NA	Ki67↓, TUNEL H score↑, CD31↓, SMA↑	30 mg/kg/day HFHC, p.o	Pelton et al., (2014)
Liver cancer	PTEN-null mice	NA	TNF-α↓, IL-1β↓, CCL2↓, F4/80-positive macrophage↓, Ki67↓, PCNA↓, VEGF↓, CD31↓, Col1a1↓, TIMP- 1↓, TGF- β↓	50 mg/kg/day. HFD, p.o	Miura et al., (2019)
	MUP-uPA mice, DEN-treated WT mice	NA	Col1a1↓, Acta2↓, Spp1↓, Pd-1L (cd274) ↓, Ctla4↓, entpd2↓, Ly6d↓, Afp↓, Gpc3↓, Birc5↓, Cd44↓	10 mg/kg/day HFHC, p.o	Ribas et al., (2021)
Pancreatic cancer	PDC-derived xenograft mouse models	Patient-derived cell (PDC)	N1C1L1↓	5 mg/day. alone, i.p	Nicolle et al., (2017)
Colorectal cancer	NPC1L1^−/−^ mice	NA	p-c-Jun↓, p-ERK↓, Caspase-1 p20↓, β-catenin↓	NA	He et al., (2015)
Renal cell carcinoma	Renca cell-derived xenograft mouse models	NA	CD8^+^ lymphocyte↑	30 mg/kg/day. alone, p.o	Wang et al., (2022)
melanoma	B16 cell-derived xenograft mouse models	NA	CD8^+^ lymphocyte↑	30 mg/kg/day. alone, p.o	Wang et al., (2022)

NA, not available; HFHC, high fat/high cholesterol diet; HFD,:high fat diet.

## Anti-Cancer Mechanisms of Ezetimibe

### Anti-Angiogenesis

**FIGURE 1:**
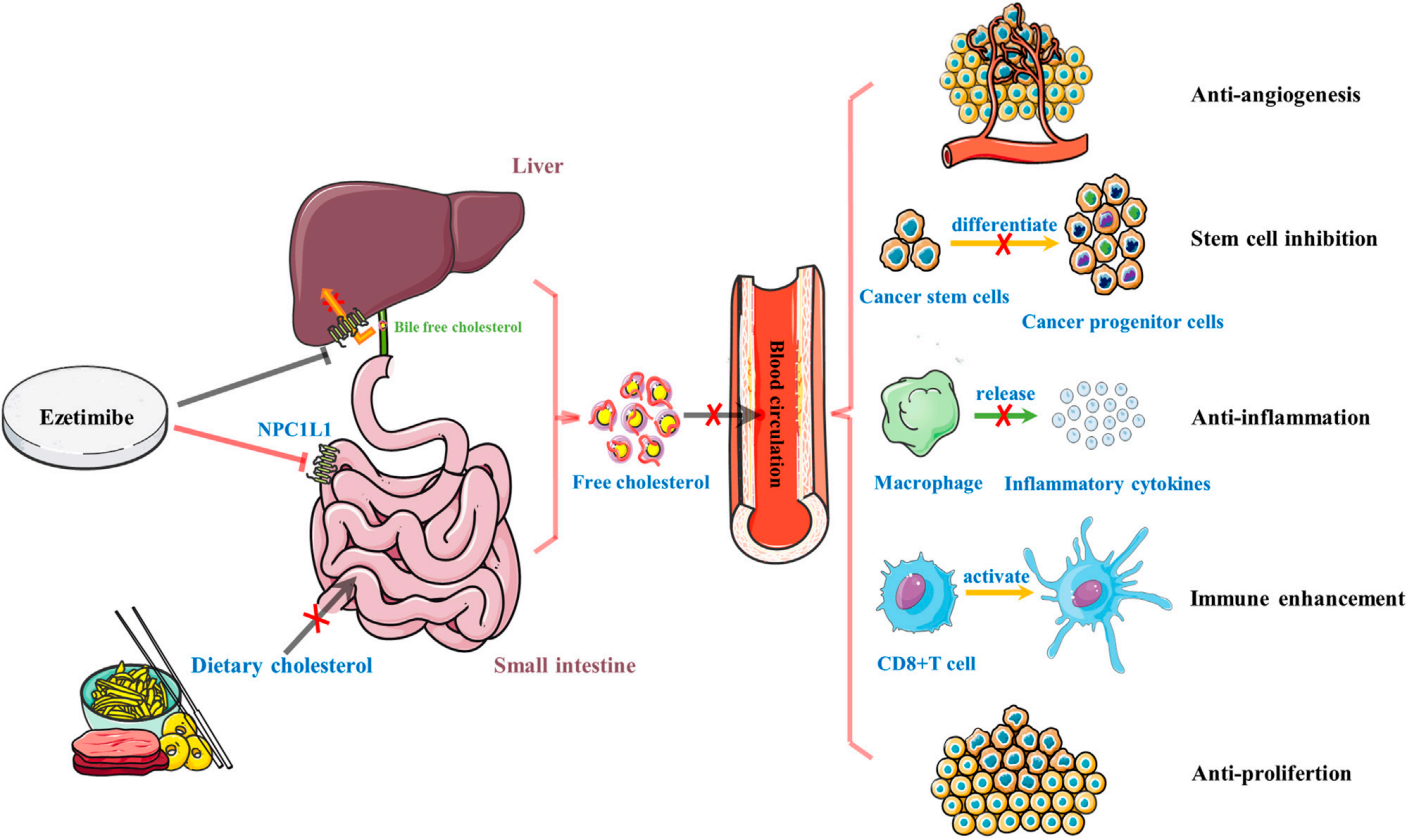
A brief summary of the role of ezetimibe in anti-cancer. Ezetimibe reduces high levels of circulating cholesterol by inhibiting the absorption of dietary cholesterol in the small intestine and biliary free cholesterol back into the liver and exhibits its anti-cancer ability through five primary mechanisms, including anti-angiogenesis, stem cell inhibition, anti-inflammation, immune enhancement, and anti-proliferation.

High fat diet (HFD) is the leading cause of high cholesterol levels in the blood, resulting in the hypercholesterolemia event, then promoting cancer progression through a variety of mechanisms (Lyu et al., 2019; Liu et al., 2021; Zhang et al., 2021). Figure 1 briefly summarizes the anticancer effect of ezetimibe. One well-established anticancer effect of ezetimibe is its anti-angiogenesis activity. Ezetimibe inhibits the germination and the growth of neovascularization. Previous studies have reported that in LNCaP human prostate cancer xenografts mice which were fed with a high fat/high cholesterol diet (HFHC), high levels of circulating cholesterol promote tumor angiogenesis by reducing the expression of angiogenesis inhibitor thrombospondin-1 (TSP-1, an effective angiogenic suppressor), and increasing the instability of vascular structure and the amount of tumor-associated microvessels density (Solomon et al., 2009; Zhang et al., 2018b). More importantly, by inhibiting CD31 (platelet endothelial cell adhesion molecule 1) and ki67, and increasing the expression of TSP-1 and SMA (smooth muscle actin, a perivascular cell marker), ezetimibe can significantly inhibit angiogenesis, promote apoptosis and prevent cell proliferation, so as to inhibit the growth of prostate tumor (Solomon et al., 2009). In addition, in breast cancer mice model, by decreasing angiogenesis, cell proliferation and elevating apoptosis, ezetimibe reduced the growth of tumors that are stimulated by the HFHC diet. Specifically, ezetimibe inhibits the expression of ki67 and CD31, and upregulates the expression of SMA (Pelton et al., 2014), supporting that hypercholesterolemia is closely associated with the progression and recurrence of breast cancer (Borgquist et al., 2017). These findings validate the direct evidence of the anticancer effects of ezetimibe, and also confirmed that cholesterol-lowering treatment can suppress angiogenesis and inhibit tumor growth. Overall, these studies illustrate that in patients with hypercholesterolemia, the lipid-lowering agents may be effective and feasible for them to prevent cancer.

Miura et al. found that in HFD-induced hypercholesterolemia and steatohepatitis-related hepatocellular carcinoma (HCC) models, ezetimibe protects mice from HCC by reducing serum and liver cholesterol levels and inhibiting angiogenesis that were induced by CD31 and VEGF (vascular endothelial growth factor) (Miura et al., 2019). Notably, inflammation cytokines (TNF-α, IL-1β, CCL2) and liver fibrosis-related makers (Col1a1, TIMP-1, TGF-β) are detected in this model. The hepatoma cell proliferation are simultaneously suppressed by ezetimibe. Interestingly, ezetimibe cannot inhibit angiogenesis in the normal diet group, suggesting that fat overload plays an important role in the anti-angiogenesis effect of ezetimibe (Miura et al., 2019). It is worth noting that in LNCaP human prostate cancer xenografts mice, no difference in the expression of pro-angiogenic factors VEGF or basic fibroblast growth factor (bFGF) is observed (Solomon et al., 2009). This may be due to the absence of NPC1L1 in the prostate. NPC1L1 is mainly expressed in liver and small intestine. Thus, the potential mechanisms of ezetimibe in the inhibition of HCC may be depended on its inhibition on HFD-induced angiogenesis, especially in HCC with hypercholesterolemia.

### Anti-Inflammation

Recently, accumulating evidence has revealed that inflammatory process driven by lipid metabolism disorder is strongly related to cancer pathogenesis and progression (Rohena-Rivera et al., 2017; Prevete et al., 2018; Riccardi et al., 2020). Several studies have determined that ezetimibe, as a potent NPC1L1 inhibitor, has the potential to regulate lipid metabolism (Tanaka et al., 2020; Xia et al., 2020) and inflammation (Kim et al., 2017; Yu et al., 2020). Apart from blocking cholesterol uptake, NPC1L1 inhibition is also involved in the regulation of other lipids, including triglyceride (TG), phospholipid (PL), high-density lipoprotein (HDL), and LDL, which can prevent HFD-induced hypercholesterolemia and fatty liver (Davies et al., 2005; Jia et al., 2010). Moreover, *in vivo* knockdown of NPC1L1 has been shown to reduce plasma lipid levels, inflammatory cell infiltrating lymphadenectasis, and colitis-associated colorectal tumors by reducing the expression of pro-inflammatory markers, including phosphorylated c-Jun (p-c-Jun), phosphorylated extra-cellular signal-regulated kinase (p-ERK), and Caspase-1 p20 (He et al., 2015). Furthermore, compared with adjacent colons, NPC1L1 knockout significantly downregulates the expression of β-catenin (He et al., 2015). β-catenin has been reported to promote colorectal tumorigenesis (Cho et al., 2020; Tian et al., 2020). Another study revealed that in guinea pig model fed with HFD, ezetimibe could suppress almost all hepatic nuclear factor kappa-B (NF-κB) activation that are induced by lipid accumulation and inflammatory response in hepatic tissue (Fraunberger et al., 2017).

Gallbladder stone, which can damage the gallbladder epithelium and initiate chronic inflammation, is the main cause of gallbladder cancer (GBC) (Lammert et al., 2016). Previous observations have shown that ezetimibe could completely prevent the occurrence and development of chronic inflammation, metaplasia and dysplasia by reducing the level of interstitial macrophage and the infiltration of polymorphonuclear cell and CD8+T cell (Rosa et al., 2020). In addition, estrogen is considered as a critical driver for gallstone formation in male prostate cancer patients who are treated with estrogen (Henriksson et al., 1989). It has been reported that ezetimibe can prevent the estrogen-mediated lithogenic actions on gallstone formation in mice, which may provide an efficacious novel strategy for the prevention of cholesterol gallstones in high-risk subjects, especially for prostate cancer patients who are exposed to high levels of estrogen (de Bari et al., 2014). Taken together, ezetimibe reduces the occurrence and development of cancer by inhibiting lipid accumulation, suppressing leukocyte infiltration and decreasing the expression of inflammatory cytokines, including p-c-Jun, p-ERK, Caspase-1 p20, and NF-κB. Additionally, ezetimibe may prevent gallbladder cancer and reduce the side effect of estrogen in the treatment of prostate cancer by reducing the formation of gallbladder stones.

### Immune Enhancement

Lipotoxicity induced by excessive cholesterol is one of the most critical drivers in the pathogenesis and progression of nonalcoholic fatty liver disease (NAFLD), which can progress to nonalcoholic steatohepatitis (NASH) and even HCC (Ioannou, 2016). As the final stage of NAFLD, HCC is the main cause of adult morbidity and mortality worldwide. NASH is the key risk factor for HCC (El-Serag and Kanwal, 2014; Forner et al., 2018). Recent reports suggest that cholesterol rather than hepatic steatosis promotes the development of NASH to HCC. Ezetimibe prevents HFHC diet-induced liver fibrosis, tumorigenesis, and NASH-driven HCC by inhibiting the intestinal absorption of dietary cholesterol and liver cholesterol content in diethylnitrosamine (DEN)-treated wild-type (WT) mice and transgenic MUP-uPA mice (Ribas et al., 2021). More specifically, the mechanism is that ezetimibe reduces the expression of involved genes in fibrogenesis (Col1a1, Acta2, and Spp1) and immune checkpoints (cd274, also known as Pd-1L, Ctla4, and entpd2), as well as the mRNA expression of HCC-related markers (Ly6d, Afp, Gpc3, Birc5, and Cd44) (Ribas et al., 2021). Zhang et al. report that ezetimibe drives serum cholesterol-lowering, promotes antitumor immunity and extenuates prostate tumor growth and metastasis by suppressing protein kinase B (Akt) phosphorylation and mammalian target of rapamycin complex 2 (mTORC2) signaling in lymphocytes, and by enhancing CD8^+^ lymphocytes memory function and tumor infiltration (Wang et al., 2022). Moreover, ezetimibe promotes fatty acid oxidation by increasing the expression of carnitine palmitoyl-transferase 1A (CPT1A) in CD8^+^ lymphocytes, which is associated to mTOR pathway and central CD8^+^ memory cells. This work also confirmed that cholesterol-lowering interventions reduce the growth of other tumors, including melanoma and renal cell carcinoma, in a CD8^+^ lymphocyte-dependent manner. It is worth noting that another study demonstrates that in streptozotocin and HFD-induced NASH-derived HCC model mouse (STAM mice), ezetimibe can suppress the progression of hepatic steatosis but ezetimibe itself alone is not strong enough to inhibit hepatic tumorigenesis (Orime et al., 2016). However, this experiment only evaluates the incidence of hepatic tumors in the 11-week early stage STAM mice models, so it cannot represent the long-term anticancer effects of ezetimibe. Therefore, more *in vitro* and *in vivo* experimental data are needed to comprehensively describe the anticarcinogenic effect and the potential molecular mechanism of ezetimibe.

### The Inhibition of Cancer Stem Cells

**FIGURE 2:**
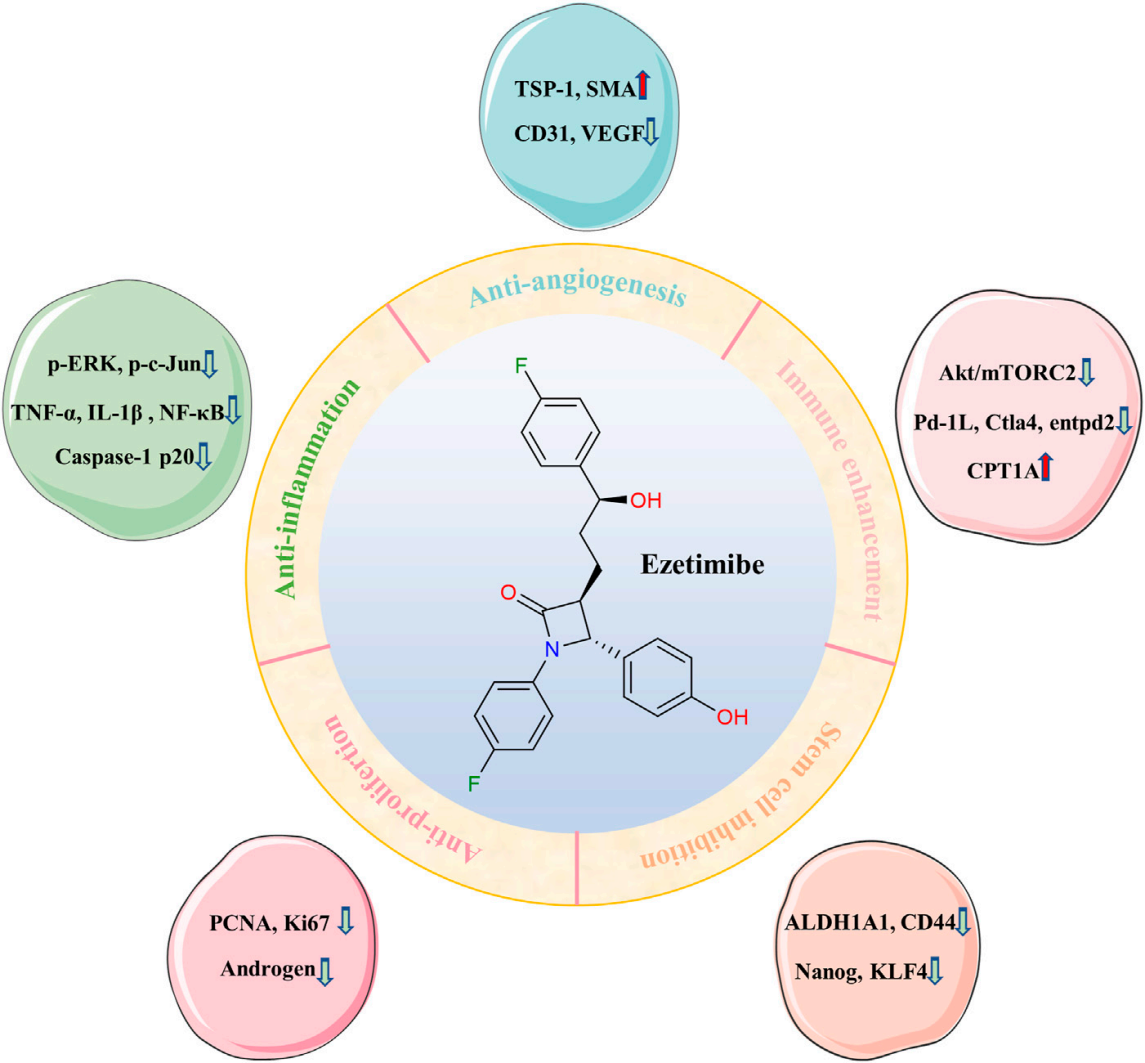
The general map of mechanisms of ezetimibe against cancer.

Ezetimibe is also involved in inhibiting cancer stem cells, which are closely related to tumor invasion and treatment resistance (Milanovic et al., 2018). In the hypercholesterolemic urinary bladder cancer (UBC) mouse model, ezetimibe significantly suppress HFHC-induced serum lipid (TC, LDL-C, and ox-LDL), and decrease the percentage of cancer cells (CK5+, CK14+, and p-STAT3+) and cancer stemness markers (ALDH1A1, CD44, KLF4, and Nanog) [1]. This work further investigates the role of hypercholesterolemia in UBC progression, and suggests that the increase of plasma ox-LDL is related to hypercholesterolemia and UBC progression, and promotes cancer stem cells via scavenger receptor B2 (CD36)/janus kinase 2 (JAK2)/signal transducer and activator of transcription 3 (STAT3) axis (Yang et al., 2021). Figure 2 shows an overview of the main anticancer mechanisms of ezetimibe.

### Other Mechanisms

Epidemiological and preclinical observations have suggested that cholesterol-lowering plays a role in reducing the development and progression prostate cancer (Alfaqih et al., 2017; Revilla et al., 2021). Cholesterol is an important precursor for *de novo* androgen synthesis (Kothandapani et al., 2021). Similarly, androgen also plays a critical role in the pathogenesis and development of prostate cancer (Dai et al., 2017). Previous researches have displayed that ezetimibe is as effective as finasteride, a potent prostatic androgen dihydrotestosterone inhibitor, in reducing prostate growth and reversing the enlarged-prostate shrinkage to normal size by decreasing circulating cholesterol levels in the hamsters model of benign prostatic hyperplasia (BPH) (Pelton et al., 2010). Lower urinary tract symptoms (LUTS) are often accompanied by BPH progression. Unlike ezetimibe, clinical studies have revealed that statins cannot inhibit LUTS in male patients with BPH (Kramer et al., 2022). Moreover, in the tumor suppressor gene phosphatase and tensin homolog (PTEN)-null prostate cancer mouse model, ezetimibe reduces plasma cholesterol, inhibits cancer cell proliferation with impaired ki67 expression, promotes tumor apoptosis, attenuates tumor androgens, and slows tumor growth (Allott et al., 2018). This is consistent with a previous study that shows ezetimibe to inhibit the growth of prostate cancer in LNCaP xenografts model (Solomon et al., 2009). These results further demonstrate the key role of ezetimibe in prostate cancer. Nevertheless, the exact molecular mechanism of ezetimibe in prostate cancer has not been fully clarified. In addition, a previous study specifically shows that circulating cholesterol-lowering therapy by ezetimibe cannot slow down the tumor growth of prostate cancer in LAPC-4 xenografts model (Masko et al., 2017). Although serum cholesterol concentrations decrease, the cholesterol levels in tumors increase significantly after ezetimibe treatment. The mechanism of drug resistance is to counteract the effect of serum cholesterol-lowering therapy through the elevation of LDLR expression. Recently, a report by Rémy Nicolle and colleagues shows that compared to NPC1L1^−/−^ tumor cells, ezetimibe could more effectively inhibit the proliferation of pancreatic tumor cells, indicating that the inhibition of NPC1L1 by ezetimibe may be an effective method for the treatment of pancreatic cancer (Nicolle et al., 2017).

## Support or Oppose the View That Ezetimibe Causes Cancer

The controversy about whether ezetimibe combined simvastatin can cause cancer comes from a 4-year clinical trial, Simvastatin Ezetimibe in Aortic Stenosis (SEAS) study (NCT00092677), with a total of 1873 patients (Rossebø et al., 2008). In this trial, compared with the placebo group, the dramatic decrease in LDL cholesterol caused by ezetimibe and lipid-lowering therapy seems to increase the incidence rate of cancer (Drazen et al., 2008). Therefore, the Food and Drug Administration (FDA) becomes more caution about the safety and effectiveness of the combination of ezetimibe and simvastatin. However, to date, a significant correlation between statins and increased cancer risk has not yet been verified by a large number of clinical data on statin therapy (Emberson et al., 2012; Ren et al., 2021). Because there is no long-term safety data from large-scale investigations at that time, so the suspicion still focused on ezetimibe. The potent cholesterol-lowering effect of ezetimibe alone or in combination with simvastatin cannot be denied. In order to validate the reliability, two clinical trials were subsequently performed. One is the Study of Heart and Renal Protection (SHARP) (NCT00125593) with 9,264 patients (mean follow-up, 2.7 years) and the other is the Improved Reduction of Outcomes: Vytorin Efficacy International Trial (IMPROVE-IT) (NCT00202878) with 11,353 patients (mean follow-up, 1.0 year). According to these two important studies, there was no credible evidence for the raised cancer risk related to ezetimibe (Peto et al., 2008). These results are consistent with a subsequent large-scale post-marketing analysis of the adverse events reported by patients with ezetimibe lone or combined with simvastatin (Alsheikh-Ali and Karas, 2009). Although there is no association between ezetimibe and the increased cancer risk in SHARP and IMPROVE-IT trials, the SEAS study indicates that ezetimibe could disturb the cancerogenic signals. The conclusion from SHARP and IMPROVE-IT trials also seem to be controversial, because they are prematurely unblinded to enable their analysis, and the follow-up time is too short (Taylor and Nissen, 2008). Unexpected and premature disclosures of the relationship between ezetimibe and increased cancer risk are also spurred at least in part by Securities and Exchange Commission (SEC) regulations (Califf et al., 2009). Also, further studies does not confirm the possible link between ezetimibe and cancer, that is, observed in the SEAS trial. The analysis of the above three clinical trials does not fully prove the hypothesis that ezetimibe will increase cancer risk due to the credibility-deficiency in ongoing trials. Even simvastatin and ezetimibe did not reduce the composite outcome of combined aortic valve events and ischemic events in patients with aortic stenosis (Rossebø et al., 2008). Such therapy reduced the incidence of ischemic cardiovascular events, but did not reduce the events associated to aortic valve stenosis. So far, this phenomenon cannot be clearly explained by convincing evidence.

After an additional 21-month follow-up study after the SEAS trial, researchers further find that ezetimibe and simvastatin do not increased the risk of cancer or related mortality compared with the control group (Green et al., 2014). Moreover, in a prospective systematic analysis of cancer incidences in IMPROVE-IT with 17,708 patients, Robert P Giugliano et al. find that simvastatin/ezetimibe therapy has no effect on cancer risk, and show that such a result might be due to the imbalance of the cancer events (Giugliano et al., 2020). In a meta-analysis of large-scale clinical trials, Costas Thomopoulos et al. demonstrate that ezetimibe/simvastatin is safe in reducing cholesterol level and will not be accompanied by changes in cancer rate (Thomopoulos et al., 2015). These results are exciting, but these is also a need for continuous monitoring of cancer outcomes during the ezetimibe treatment. Recently, Kobberø Lauridsen et al. reveal that the long-term genetic inhibition of NPC1L1 (encoding the target of ezetimibe) did not increase the cancer risk of 67,257 patients, and showed that the prolonged treatment with ezetimibe is less likely to increase the risk of cancer (Kobberø Lauridsen et al., 2017). A subsequent Mendelian randomization study has indeed agreed with this genetic study (Nowak and Ärnlöv, 2018). Table 2 briefly summarizes human studies of ezetimibe on cancer risk. The preclinical results of ezetimibe used to evaluate whether ezetimibe is carcinogenic show that ezetimibe has no structural alarm of genotoxicity or carcinogenicity. More importantly, ezetimibe is not carcinogenic in the standard 2-year bioassays of mice and rats (Halleck et al., 2009). In NPC1L1 knockout mice, no evidence of ezetimibe-driven tumor is observed, which do not support the hypothesis of SEAS trial. Overall, after the conclusion of SEAS trial is released, more and more researchers try to prove that ezetimibe does not increases the risk of cancer. However, the experimental data indicating whether ezetimibe is short-term or long-term carcinogeic and whether it has anticancer effect are still limited. Therefore, more preclinical experiments and clinical trials with longer follow-up duration are needed. To date, there are no clinical studies of ezetimibe alone in the treatment of patients with cancer, and its clinical efficacy on cancer remains unclear. However, numerous preclinical studies have demonstrated some significant therapeutic effects of ezetimibe in cancer models *in vivo and in vitro*. Currently, there are no studies reporting the clinical dose safety of ezetimibe in cancer patients, and its efficacy on cancers and safety in experimental animals are still in preclinical studies. Further clinical trials are required to confirm the efficacy and safety of ezetimibe in cancer patients.

**TABLE 2:** Clinical evaluation of ezetimibe on cancer risk.

Trial	Patients	Follow-up	Treatment arms	Main results	References
SEAS	1873	4.0-year	ezetimibe 10 mg + Simvastatin 40 mg	ezetimibe/simvastatin seemed to increase cancer risk	Rossebø et al., (2008)
SHARP	9,264	2.7-year (mean)	ezetimibe 10 mg + Simvastatin 20 mg	there was no association between ezetimibe/simvastatin and the increased cancer risk	Peto et al., (2008)
IMPROVE-IT	11,353	1.0-year (mean)	ezetimibe 10 mg + Simvastatin 40 mg		Peto et al., (2008)
Anders et al	1,359	21-month	ezetimibe 10 mg + Simvastatin 40 mg	ezetimibe/simvastatin did not increased cancer risk	Green et al., (2014)
Robert P et al	17,708	6.0-year (mean)	Simvastatin 40 mg alone or ezetimibe 10 mg + Simvastatin 40 mg	ezetimibe/simvastatin had no effect on cancer risk	Giugliano et al., (2020)

## Perspectives

Collectively, N1C1L1-mediated exogenous cholesterol uptake is essential for cholesterol homeostasis, and high levels of circulating cholesterol is an important inducement for the occurrence and development of cancer. Therefore, ezetimibe, a drug that reduces cholesterol by inhibiting NPC1L1, is considered to be an effective and feasible therapeutic approach for cancer.

In the past decade, ezetimibe has shown its potential in the treatment and prevention of a variety of cancers. It is capable of inhibiting angiogenesis, inducing apoptosis, suppressing tumor proliferation, inhibiting inflammation and stemness, and potentiating immune. However, the specific mechanism of ezetimibe as a tumor inhibitor is not yet completely understood. The studies on the anticancer effect of ezetimibe mainly focus on the cell-based and animal-based preclinical models. Many studies have shown that ezetimibe has no inhibitory effect on cancer cells; instead, it mainly depends on reducing exogenous cholesterol to suppress tumor growth. Preclinical and clinical studies on the antitumor effect of ezetimibe are still very limited, and the role of ezetimibe in many cancer types has not been fully studied. Another relevant limitation is the lack of specialized epidemiologic and clinical studies to support its role in cancer. Although previous studies have shown that ezetimibe combined with simvastatin increases the risk of cancer, the conclusion of SEAS trial seems unreliable. More and more evidence supports the role of ezetimibe in cancer treatment and prevention, especially in cancers with high serum cholesterol levels. Moreover, no studies have so far shown significant toxicological side-effects of ezetimibe in cancer treatment. Nonetheless, more large-scale epidemiological studies and clinical trials with a longer follow-up time are needed to understand the safety and efficacy of ezetimibe in cancers. Taken together, we highlight the potential of ezetimibe as a future personalized cancer therapeutic agent.

As one of the most common lipids that support the growth and proliferation of cancer cells, cholesterol is involved in regulating the rigidity, fluidity, and permeability of the lipid bilayer membrane. It is a precursor of vitamin D, bile acid and steroid hormone (such as androgen) (Sezgin et al., 2017). One of the fundamental characteristics of cancer cells is the enhanced cholesterol metabolisms, such as high cholesterol absorption and *de novo* cholesterol synthesis, which contributes to cancer progression. Additionally, with regard to immune regulation, Boliang Li’s team has confirmed that acyl-coenzyme A cholesterol acyltransferase 1 (ACAT1)-deficient CD8 + T cells are superior to wild-type CD8^+^ T cells in controlling the growth and metastasis of melanoma in mice (Yang et al., 2016). ACAT1 is a key regulator of cholesterol modification to cholesteryl ester (CE), which is stored in lipid droplets. Therefore, targeted therapy for cholesterol metabolism is considered to be a practical method for cancer treatment. However, in order to maintain the essence, that is, the chronic proliferation of cancer cells themselves, cancer cells may have the specific ability to utilize cholesterol, regardless of the absorption of cholesterol from the diet and *de novo* synthesis of cholesterol, which are blocked by ezetimibe and statins, respectively. This “specific ability” may be a real killer, which needs a lot of research to confirm.

## Glossary

ACAT1: acyl-coenzyme A cholesterol acyltransferase 1
Akt: protein kinase B
bFGF: basic fibroblast growth factor
BPH: benign prostatic hyperplasia
CD31: platelet endothelial cell adhesion molecule 1
CD36: scavenger receptor B2
CE: cholesteryl ester
CPT1A: carnitine palmitoyl-transferase 1A
CVD: cardiovascular disease
DEN: diethylnitrosamine
ERC: endocytic recycling compartment
FDA: food and drug administration
GBC: gallbladder cancer
HCC: hepatocellular carcinoma
HDL: high-density lipoprotein
HFD: high fat diet
HFHC: high fat/high cholesterol diet
HMGCR: HMG-CoA reductase
IMPROVE-IT: improved reduction of outcomes: vytorin efficacy international trial
JAK2: janus kinase 2
LDL: low-density lipoprotein
LDL-C: LDL cholesterol
LDLR: LDL receptor
LUTS: Lower urinary tract symptoms
mTORC2: mammalian target of rapamycin complex 2
NAFLD: nonalcoholic fatty liver disease
NASH: nonalcoholic steatohepatitis
NF-κB: nuclear factor kappa-B
NPC1: Niemann–Pick type C1
NPC1L1: sterol transporter Niemann-Pick C1-Like 1
ox-LDL: oxidized LDL
PCSK9: proprotein convertase subtilisin/kexin type 9
p-c-Jun: phosphorylated c-Jun
p-ERK: phosphorylated extra-cellular signal-regulated kinase
PL: phospholipid
PTEN: phosphatase and tensin homolog
SEAS: simvastatin ezetimibe in aortic stenosis
SEC: securities and exchange commission
SHARP: study of heart and renal protection
SMA: smooth muscle actin
STAT3: signal transducer and activator of transcription 3
TG: triglyceride
TSP-1: thrombospondin-1
UBC: urinary bladder cancer
VEGF: vascular endothelial growth factor
